# Genome sequence of *Lactobacillus johnsonii* XZ17, isolated from a C57BL/6J mouse lung

**DOI:** 10.1128/mra.00555-24

**Published:** 2024-08-20

**Authors:** Keerthikka Ravi, Gina J. Oh, Bethany B. Moore, Gary B. Huffnagle, Xiaofeng Zhou

**Affiliations:** 1 Department of Molecular, Cellular, and Developmental Biology, University of Michigan, Ann Arbor, Michigan, USA; 2 Department of Microbiology and Immunology, University of Michigan, Ann Arbor, Michigan, USA; 3 Division of Pulmonary and Critical Care Medicine, Department of Internal Medicine, University of Michigan, Ann Arbor, Michigan, USA; 4 Mary H. Weiser Food Allergy Center, University of Michigan, Ann Arbor, Michigan, USA; Department of Biological Sciences, Wellesley College, Wellesley, Massachusetts, USA

**Keywords:** lactic acid bacteria, lung microbiome, immunomodulation, BMT mice

## Abstract

*Lactobacillus johnsonii* XZ17 was isolated from the lung homogenate of a healthy C57BL/6J mouse. XZ17 is diminished in the lungs of syngeneic bone marrow-transplanted recipient mice. Long-read sequencing of XZ17 yielded a single genome of 1,948,140 bp, with a GC content of 34.64%.

## ANNOUNCEMENT

*Lactobacillus johnsonii* is a lactic acid bacterium associated with the healthy gut of many vertebrate hosts (1, 2). As a probiotic, this species has several health-promoting effects, like inhibition and exclusion of pathogens (3–5) and host immunomodulation (6, 7). Here, we present the genome sequence of *L. johnsonii* strain XZ17 isolated from the mouse lung. Notably, *L. johnsonii* XZ17 abundance decreases in mouse lungs following bone marrow transplantation (8, 9).

A healthy C57BL/6J mouse, housed in a specific pathogen-free animal research facility at the University of Michigan, was euthanized by CO_2_. The mouse lung was aseptically isolated, homogenized, and cultured on de Man, Rogosa, and Sharpe (MRS) agar for 24 hours at 37°C under aerobic conditions. Several pure cultures were established by re-streaking and subsequently maintained as frozen stocks. Sequencing the 16S rRNA revealed that all isolates were identical and exhibited 100% homology with the known 16S rRNA sequence of *L. johnsonii* (GeneBank CP091981.1). One isolate, XZ17, was selected for comprehensive genomic sequencing. For isolating high molecular weight (HMW) DNA, an overnight bacterial culture of XZ17 in MRS broth was grown aerobically at 37°C (16–18 hours of incubation), and Nanobind HMW DNA extraction kit (Circulomics) with RNase treatment was used for DNA isolation.

Sequencing libraries were prepared using the ligation sequencing kit (SQK-LSK109) and selecting DNA fragments of all sizes with the short fragment buffer (SFB), according to the Oxford Nanopore Technologies library preparation protocol. The libraries were sequenced on an R9.4.1 ﬂow cell for 3 hours using a MinION sequencer (Oxford Nanopore Technologies). Guppy v6.0.7, in high-accuracy mode, was used for base calling. A total of 24 Mb was obtained after base calling, distributed across 7,712 reads, with a maximum length of a read at 62,782 bp and an *N*_50_ value of 8,282 bp. The genome sequences were assembled *de novo* as previously described (10). *De novo* assembly was performed using Flye v2.9 (11) with default parameters. The assembly was further polished through two rounds of Racon v1.5.0 (12) using the default parameters for all except score for matching and mismatching bases (--match 8 --mismatch -6). A final round of polish was carried out using Medaka v1.2.3 (13), using default parameters. XZ17 (genome coverage, 35×) was finally assembled into one contig of length 1.9 Mb. CheckM analysis (v1.2.2) (14), using taxonomic workflow for *L. johnsonii* species, reported a completeness of 93.65% and a contamination rate of 0.83%. The XZ17 genome is 1,948,140 bp and has a GC content of 34.64%. The genome of *L. johnsonii* is characterized by low GC content, with an average of 34.47% observed across 89 strains (15).

Sequence annotation for the strain was performed using the NCBI Prokaryotic Genome Annotation Pipeline (PGAP) (v6.7) (16). The annotation predicted 1,879 genes, 1,780 coding sequences, 68 pseudogenes, 78 tRNAs, 6 rRNAs (5S, 16S, and 23S), and 3 noncoding RNAs.

## Data Availability

All data are available under BioProject accession number PRJNA1095000 and BioSample accession number SAMN40710645. The genome was deposited in GenBank (CP151183.1), and the nanopore base-called data were submitted to SRA (SRR28520834).
